# NAD^+^-Metabolizing Ectoenzymes in Remodeling Tumor–Host Interactions: The Human Myeloma Model

**DOI:** 10.3390/cells4030520

**Published:** 2015-09-17

**Authors:** Alberto L. Horenstein, Antonella Chillemi, Valeria Quarona, Andrea Zito, Ilaria Roato, Fabio Morandi, Danilo Marimpietri, Marina Bolzoni, Denise Toscani, Robert J. Oldham, Massimiliano Cuccioloni, A. Kate Sasser, Vito Pistoia, Nicola Giuliani, Fabio Malavasi

**Affiliations:** 1 Laboratory of Immunogenetics, Department of Medical Sciences, University of Torino, Torino 10126, Italy; E-Mails: antonella.chillemi@unito.it (A.C.); valeria.quarona@unito.it (V.Q.); andrea.zito@unito.it (A.Z.); 2 CeRMS, University of Torino, Torino 10126, Italy; E-Mail: roato78@libero.it; 3 Laboratory of Oncology, Istituto Giannina Gaslini, Genova 16148, Italy; E-Mails: fabiomorandi@ospedale-gaslini.ge.it (F.M.); danilomarimpietri@ospedale-gaslini.ge.it (D.M.); vitopistoia@ospedale-gaslini.ge.it (V.P.); 4 Myeloma Unit, Department of Clinical and Experimental Medicine, University of Parma, Parma 43126, Italy; E-Mails: marina.bolzoni@unipr.it (M.B.); denise.toscani@nemo.unipr.it (D.T.); nicola.giuliani@unipr.it (N.G.); 5 Antibody & Vaccine Group, Cancer Sciences Unit, Faculty of Medicine, University of Southampton, Southampton General Hospital, Southampton SO16 6YD, UK; E-Mail: Robert.Oldham@soton.ac.uk; 6 School of Biosciences and Veterinary Medicine, University of Camerino, Camerino 62032, Italy; E-Mail: massimiliano.cuccioloni@unicam.it; 7 Janssen Research & Development, LLC, Springhouse, PA 19477, USA; E-Mail: ksasser1@its.jnj.com

**Keywords:** NAD^+^, CD38, ectoenzymes, adenosine, multiple myeloma, Daratumumab

## Abstract

Nicotinamide adenine dinucleotide (NAD^+^) is an essential co-enzyme reported to operate both intra- and extracellularly. In the extracellular space, NAD^+^ can elicit signals by binding purinergic P2 receptors or it can serve as the substrate for a chain of ectoenzymes. As a substrate, it is converted to adenosine (ADO) and then taken up by the cells, where it is transformed and reincorporated into the intracellular nucleotide pool. Nucleotide-nucleoside conversion is regulated by membrane-bound ectoenzymes. CD38, the main mammalian enzyme that hydrolyzes NAD^+^, belongs to the ectoenzymatic network generating intracellular Ca^2+^-active metabolites. Within this general framework, the extracellular conversion of NAD^+^ can vary significantly according to the tissue environment or pathological conditions. Accumulating evidence suggests that tumor cells exploit such a network for migrating and homing to protected areas and, even more importantly, for evading the immune response. We report on the experience of this lab to exploit human multiple myeloma (MM), a neoplastic expansion of plasma cells, as a model to investigate these issues. MM cells express high levels of surface CD38 and grow in an environment prevalently represented by closed niches hosted in the bone marrow (BM). An original approach of this study derives from the recent use of the clinical availability of therapeutic anti-CD38 monoclonal antibodies (mAbs) in perturbing tumor viability and enzymatic functions in conditions mimicking what happens *in vivo.*

## 1. Introduction

The social life of cells is regulated by a variety of mechanisms ranging from soluble factors to physical crosstalk among homo- or hetero-typic cells. These soluble factors and adhesion-mediated effects are, in turn, regulated by the cellular environment. Closed environmental systems such as niches or microenvironments provide the ideal setting for soluble factors and adhesion effects to acquire new and different functions, sometimes even antithetic to the ones they perform in open systems, such as the blood stream.

Blood supply, immune evasion, and access to supplementary sources of energy are of key relevance for tumor cell viability. In the most common strategy for survival, normal molecules are hijacked and adapted for the tumors’ own use. Phylogenetic evidence indicates that the most ancient and rudimentary interactions between cell and environment are ruled by monomorphic soluble factors regulating crucial signals for the life and death of cells. Central to this plot is the concerted action of the extracellular nucleotides and their receptors, which are involved in the regulation of metabolism. Nicotinamide adenine dinucleotide (NAD^+^) is one of these players.

This work focuses on NAD^+^ and on CD38, the main NAD^+^-consuming ectoenzyme. The study design adopted is original, since the majority of the translational conclusions are derived from observations validated in samples of human multiple myeloma.

One advantage of using multiple myeloma (MM), a neoplastic expansion of plasma cells, as a model is that MM cells express high levels of surface CD38. In addition, myeloma cells generally live in closed environments located in the bone and CD38 expression by these cells may be functional to growth and escape strategies. Finally, the effects induced by therapeutic anti-CD38 monoclonal antibodies provide proof-of-concept that human multiple myeloma might use CD38 and its main substrate NAD^+^ in generating local tolerance.

The findings of this study may provide basic scientists with valuable clues as to the role of ectoenzymes *in vitro* and make it possible for clinicians to improve *in vivo* therapy through the use of anti-CD38 reagents.

## 2. Premises

### Biogenesis of NAD^+^

Nobel laureates Harden, von Euler-Chelpin, and Warburg contributed in the early 20th century to the discovery and definition of the structure and key metabolic functions of NAD^+^ [1]. NAD^+^ is an essential cellular metabolite involved in a wide range of cellular processes, such as energy production, reductive biosynthesis, and calcium homeostasis [2]. In addition, NAD^+^ is an enzymatic substrate. Despite evidence that NAD^+^ levels influence health span and, in some cases, lifespan, NAD^+^ cannot cross the cell membrane due to its nature as a polar compound [3].

**Figure 1:**
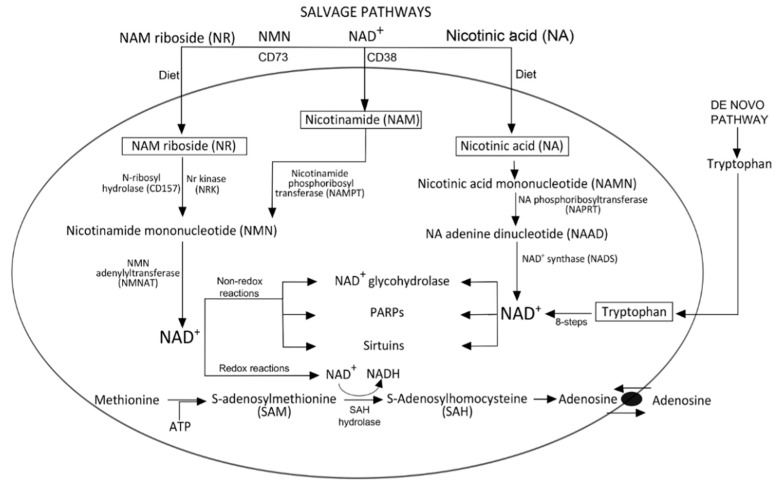
Pathways for NAD^+^ biogenesis and NAD^+^-consuming enzymes. Cellular NAD^+^ is synthesized either from dietary tryptophan or nicotinic acid and nicotinamide (referred to as nicotinic acid, vitamin B3, or niacin). Extracellular NAD^+^ can also be broken down by CD38 to produce nicotinamide (NAM) or nicotinamide mononucleotide (NMN), which can be further broken down to nicotinamide riboside (NR) in a reaction catalyzed by CD73. NR can enter cells through a nucleotide transporter, where it can participate in intracellular NAD^+^ biogenesis. NR is converted to NMN by nicotinamide riboside kinases (NRK) or by CD157 in the cytoplasm. NAM is converted by nicotinamide phosphoribosyltransferase (NAMPT) to NMN. Both pathways merge at the step of NMN formation, which is further converted to NAD^+^ by the action of NMN adenylyltransferase (NMNAT). Nicotinic acid (NA) is converted to NA mononucleotide (NAMN), NA adenine dinucleotide (NAAD), and then NAD^+^. NAD^+^ is also used as a cofactor of S-adenosylhomocysteine (SAH) hydrolase for the generation of intracellular adenosine. These reactions are not accompanied by any net consumption of NAD^+^. Conversely, a net loss of NAD^+^ is associated with enzymatic reactions that take place during ADP-ribose formation (NAD^+^ glycohydrolase), polyADP-ribosylation (PARPs), and the de-acetylation of proteins (Sirtuins).

Several metabolic routes lead to NAD^+^ synthesis from four different precursors. In detail, the dinucleotide may be obtained from (i) tryptophan as the *de novo* pathway, while (ii) nicotinamide (NAM), (iii) nicotinic acid (NA), and (iv) nicotinamide riboside (NR) represent elements of salvage pathways [4,5]. The *de novo* synthesis from L-tryptophan (obtained from the diet) is a complex 8-step enzymatic process, which likely shifts the balance to more economical 2-3 step enzymatic pathways to generate NAD^+^. One of these originates from dietary niacin (consisting of NAM and NA), which is recycled as NAD^+^ precursor by means of a salvage pathway (Figure 1). NAM is converted to NAM mononucleotide (NMN) by the NAM phosphoribosyltransferase (NAMPT) enzyme [6]. The enzyme is reported as present inside and outside the cells: the extracellular form also acts as a cytokine (pre-B cell colony-enhancing factor, PBEF), better known as visfatin [7]. NAMPT has the function to convert NAM to NAD^+^, thus lowering NAM and increasing NAD^+^ levels. In addition to its role as a rate-limiting step, NAMPT is an important regulatory component of the NAD^+^-consuming enzymes functioning inside the cells. The functions controlled include DNA repair by poly ADP-ribose polymerase (PARP) and gene expression by sirtuins. After NAMPT reaction, the NMN product is converted to NAD^+^ by nicotinamide mononucleotide adenylyltransferase (NMNAT), which condenses the adenylyl moiety to NMN [5].

A salvage route to generate NAD^+^ was recently described starting from the precursor NAM riboside (NR). NR is markedly less polar than NAD^+^ and treatment with NR increases cellular NAD^+^ levels [3,8]. As a salvageable precursor of NAD^+^, NR is phosphorylated to NMN by NR kinases (NRK), after which NMNAT catalyzes its conversion to NAD^+^ [5]. Like NA, this pathway is active in humans. NR hydrolysis is also mediated by CD157, a member of the CD38/NAD^+^-glycohydrolase family, which binds NR as a substrate better than NAD^+^ [8]. In fact, the CD157-catalyzed reaction with NR generates NAM as a product with high affinity: the 6 nM K_m_ value for hydrolysis was found to be >100,000-fold higher than that for other nucleotides [9]. CD157 is therefore confirmed as a weak NAD^+^-glycohydrolase, adding new perspective to this pathway as a potential event for therapeutic modulation in NAD^+^-dependent metabolism.

## 3. NAD^+^ Degradation

### 3.1. Role of NAD^+^ as Cofactor

The role of cofactor in oxidoreductases was attributed to NAD^+^ in 1935 [10] in reactions catalyzed by these enzymes. NAD^+^ undergoes oxidation-reduction cycles resulting in transformation of NAD^+^ to NADH and vice versa. As a cofactor, intracellular NAD^+^ is involved in the synthesis of the nucleoside adenosine (ADO). As depicted in Figure 1, ADO generation involves the hydrolysis of methionine by S-adenosylhomocysteine (SAH), the demethylated product of S-adenosylmethionine (SAM). Using NAD^+^ as a cofactor, the enzyme SAH hydrolase is responsible for this hydrolytic step. Together with the tight regulation of its biosynthesis and degradation, the co-enzymatic activity of NAD^+^ meets all the basic requirements for acting as a metabolic sensor.

### 3.2. Role of NAD^+^ as a Precursor for Additional Reactions

Intracellular NAD^+^ homeostasis is the result of the balance between a number of NAD^+^-cleaving reactions and NAD^+^ biosynthetic routes. NAD^+^ is consumed by three major families of enzymes: PARPs, sirtuins and NAD^+^-glycohydrolases [11]. Accordingly, NAD^+^ serves as (i) a substrate for post-translational protein modifications (mono- or poly-ADP-ribosylation), (ii) an acceptor for acetyl groups during de-acetylation reactions, and (iii) a precursor for signaling molecules. A common feature of these non-redox reactions is that the substrate NAD^+^ donates its ADPR group, breaking the glycosidic bond linking NAM moiety to ribose moiety, which disassembles the parent NAD^+^ molecule. The ADPR resulting from these reactions is bound to an acceptor protein (ADP-ribosylation) or to an acetyl group (de-acetylation), while the released NAM is used for NAD^+^ biogenesis.

During protein mono(ADP-ribosyl)ation, the ADPR from NAD^+^ is transferred to a specific amino acid of the acceptor protein by ART enzymes [12]. Poly(ADP-ribosyl)ation involves transfer of several ADPR molecules from NAD^+^ to an acceptor protein by PARP enzymes, leading to formation of branched polymers [13]. During protein de-acetylation, the ADPR derived from NAD^+^ is bound to the acetyl group of a lysine of the target protein, generating O-acetyl-ADP-ribose and NAM and de-acetylating the protein. This reaction is catalyzed by sirtuins, a class of proteins that have either histone deacetylase or mono(ADP-ribosyl) transferase activity [14]. By using NAD^+^ as a substrate, these enzymes are able to modulate intracellular NAD^+^ and the NAM by-product levels, as reviewed [15]. The NAD^+^-cleaving activity of these enzymes indirectly affects NAD^+^ bioavailability: the consequence is an effect on the NAD^+^ outside the cell.

## 4. Extracellular NAD^+^ Metabolism

Nucleotides have a second life in the extracellular environment as intercellular communicators and signal transducers [16]. From an evolutionary point of view, nucleotides are among the most ancient molecules with biological activity [17] and are used by living cells for multiple purposes. The pool of extracellular nucleotides include NAD^+^, whose metabolism is ruled by cell surface proteins endowed with an enzymatic domain (*i.e.*, ectoenzymes). Such ectoenzymes work in concert to disassemble extracellular nucleotides, which can only be extruded from inside the cells by active channeling mechanisms or by microvesicles.

The concentration of NAD^+^ in human plasma ranges between 50 and 100 nM [18]. These levels are the result of a balance between the opposing processes of dinucleotide release from the cells and its enzymatic degradation. NAD^+^ levels in specific tissue districts may be significantly higher than those observed in plasma, especially during inflammation. Information about NAD^+^ levels in selected human pathology models is quite recent and, thus far, incomplete.

The main extracellular NAD^+^-consuming enzyme is CD38, a type II glycosylated protein with a single transmembrane domain near its N-terminus [19,20]. CD38 was first identified as a cell activation marker and later characterized as a receptor and a signaling molecule. CD38 was then implicated in the pathogenesis of different diseases, prevalently those affecting the hematopoietic lineage [21]. Results obtained with *CD38* knock-out mice indicate that CD38 plays a key role in the release of oxytocin, a hormone involved in the control of various aspects of human behavior, in addition to parturition and lactation [22].

CD38 was also shown to be an ectoenzyme by virtue of a marked similarity with the soluble enzyme ADP-ribosyl cyclase purified from the mollusk *Aplysia*. CD38 has the capacity to produce cyclic ADP-ribose (cADPR), ADP-ribose (ADPR), and nicotinic acid adenine dinucleotide phosphate (NAADP), by the concerted action of its NAD^+^ glycohydrolases, ADPR cyclase, and cADPR hydrolase ectoenzyme activities [19], as part of a second messenger system.

The ability to use NAD^+^ as a substrate led to the postulate that CD38 acts as a metabolic sensor that limits the duration of NAD^+^-signaling in the extracellular compartment. The activity of CD38 for a given extracellular NAD^+^ level is defined by the Michaelis constant, K_m_, for the reaction. This constant describes the NAD^+^ concentration when the reaction rate is half of the maximum during NAD^+^ excess. CD38 displays a K_m_ for NAD^+^ in the low micromolar range (1–5 µM) [23]. Under normal homeostatic conditions, CD38 is expressed at low levels, whereas extracellular NAD^+^ is in limited supply (≈0.1 µM): therefore, small quantities of derived metabolites are produced after NAD^+^ disassembling by CD38. However, upon inflammation, tissue damage, or the growth of tumors, the concentration of extracellular NAD^+^ increases, and may reach concentrations of 5–10 µM, levels that exceed the K_m_ of CD38. Thereafter, CD38 protein expression, and thus ectoenzymatic activity, is up-regulated in the presence of increased extracellular NAD^+^, generating Ca^2+^ second messengers. In this respect, it is important to note that the stoichiometry of the reaction catalyzed by CD38 involves a massive amount of NAD^+^ (≈100 molecules) to yield a single cADPR [24]. Whether and how the second messenger enters the cells to elevate the cytosolic calcium is a matter of debate. However, recent topological studies have described the enzymatic activity of this transmembrane protein as both extra- (type II) and intra- (type III) cellular, the latter with an inward orientation of the active site of the enzyme [25]. According to this view, CD38 would be a critical regulator of intracellular NAD^+^, with the cytosolic production of Ca^2+^-mobilizing second messengers.

Besides acting as a substrate for CD38, NAD^+^ may also work as a cytokine, eliciting rapid functional responses through binding to specific purinergic type 2 (P2) receptors. The signaling cascade and the outcome differ according to cell type and environment. Other vital functions are controlled by NAD^+^, including proliferation, migration, and release of prostaglandin E2 and cytokines [26].

## 5. CD38 and Generation of Adenosine

The environment around a tumor is characterized by high levels of extracellular nucleotides that are metabolized through a sequential action of ectoenzymes. These catabolic activities may lead to the local production of adenosine (ADO), a nucleoside involved in the control of inflammation and immune responses.

A previously unexplored enzymatic pathway was recently characterized as an alternate route to producing extracellular ADO. This axis hinges around the nucleotide-metabolizing ectoenzymes NAD^+^-glycohydrolase CD38, the ecto-nucleotide pyrophosphatase/phosphodiesterase CD203a (also known as PC-1), and the 5′-ectonucleotidase CD73 [27]. This pathway may flank, synergize, or bypass the canonical catabolic pathway mediated by the ectonucleoside nucleoside triphosphate diphosphohydrolase-1 CD39. ADO is the final product of these reactions, and can in turn bind to unique purinergic P1 receptors and elicit signals through modulating cAMP levels. ADO, NAM, and phosphate can be taken up by cells and used for the regeneration of NAD^+^. The adenosinergic network takes part in the regulation of the signals mediated by the P2 and P1 receptors, and at the same time is an efficient scavenging system.

A possibility not fully explored is that the presence of extracellular NAD^+^-metabolizing enzymes might impact on intracellular NAD^+^ levels. This would imply involvement of the intracellular NAD^+^-consuming enzymes (e.g., sirtuins and PARPs) that regulate vital cellular processes [28].

**Figure 2:**
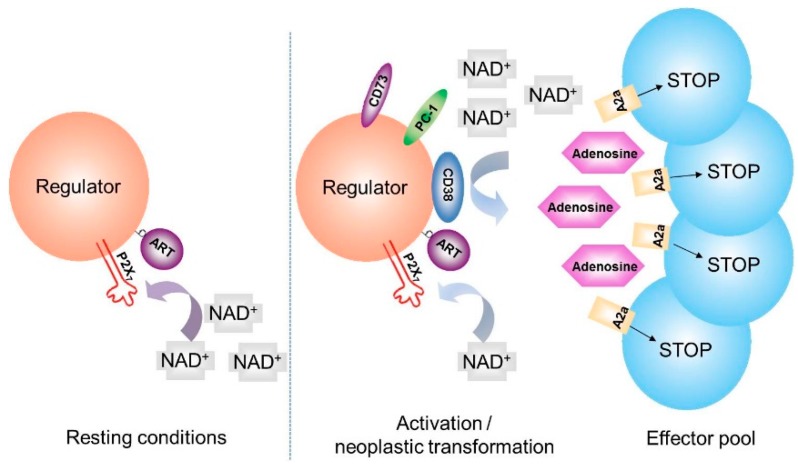
Hypothetical roles of CD38 in immunomodulatory and suppressor cell populations.

The definition of the immunomodulatory functions of NAD^+^ is currently in progress. So far, NAD^+^ appears to be a critical element in the immune system, influencing multiple and complex processes. Indeed, extracellular NAD^+^ induces multiple responses by immune cells through different ectoenzymatic activities. These include (i) the conversion by CD38, with subsequent influx of cADPR and binding to ryanodine receptors to mobilize intracellular Ca^2+^ [19] and (ii) another event mediated by mono-ADP-ribosyltransferases, which transfer ADPR-moiety of NAD^+^ to target proteins. The ART-2 isoform ADP-ribosylates specific P2 receptor in murine immune cells [29], thereby influencing phenotype and functions of regulatory T (Treg) cells. ART-2 isoform is, however, not annotated in the human genome. The subtype P2X7 receptor is a critical target of ART-2, whose ADP-ribosylation causes activation and apoptotic cell death (NAD^+^-induced cell death, NICD) (Figure 2) [30]. P2X7 is expressed by naive/resting T lymphocytes: a consequence could be that P2X7-mediated NICD specifically marks this population, becoming a homeostatic mechanism for Treg [29,31]. However the precise role of NICD in humans remains elusive. The influence on myeloid derived suppressor cells (MDSC) is still under investigation [32].

## 6. NAD^+^ Balance in Cancer: A Working Hypothesis Confirmed in a Human Myeloma Model

By virtue of its primordial nature, NAD^+^ metabolism works in a coordinated network that may be subverted by the tumor for its own use and advantage. CD38 has played a crucial role in the story of human multiple myeloma [33]. In the last decades, CD38 was considered as an activation marker, a label that protected it from sinking into oblivion. As such, it was extensively used in clinical routine and also in studies focused on human leukemias or HIV infection. In juxtaposition to the definition of CD38 as an activation marker, was the finding that plasma cells, terminally differentiated elements of the B lineage, express the highest levels of surface CD38. The role of CD38 during plasma cell differentiation is still unclear.

However, the fact that CD38 was prevalently analyzed by groups with an immunological background may have biased the study. The results obtained supported the view that the molecule is a receptor and an adhesion molecule [19]. Interest in the molecule was re-fueled by the finding of a striking similarity between a cyclase, an enzyme purified from *Aplysia californica*, and the sequence of human CD38, despite their phylogenetic separation of over 900 million years [34].

On the whole, these observations suggest that CD38 is a pleotropic molecule endowed with the ability to adhere to heterotypic cells, to transduce activatory signals involved in the control of Ca^2+^, and to act as an ectoenzyme.

CD38 was successively selected by pharma companies as an ideal target for treating human multiple myeloma with specific monoclonal antibodies (mAbs), also in virtue of its favorable expression during ontogenesis. Indeed, early hematological precursors are CD38^−^. Furthermore, CD38 expression is maintained in spite of the genomic differences marking myeloma cells. A more articulated hypothesis proposed that CD38 is not a simple marker. Because of its quantitative expression, CD38 might represent a ruler of NAD^+^ levels inside the niche where the myeloma grows. As a consequence, the enzymatic activities of this environment may lead to significant production of ADO. The outcome of the actions driven by ADO may be the generation of an environment that is extremely favorable to tumor survival in areas that are difficult to reach with drugs. This outcome may also be the result of cooperation among the different cells making up the microenvironment. Still awaiting confirmation was the hypothesis that the metabolism of ADO may influence regulatory cells, hence contributing to the silencing of immune effectors.

## 7. Ectoenzymes in the Myeloma Niche

While the expression of CD38 by human myeloma cells has been long-established, the molecule’s role in the production of ADO is a less familiar concept. Our laboratory confirmed the hypothesis that the canonical CD39 (ectonucleoside triphosphate diphosphohydrolase-1)/CD73 (5′-nucleotidase) pathway related to the conversion of ATP may be flanked by another set of surface molecules leading to the same product, but using NAD^+^ as a starting substrate. This finding was confirmed in T leukemia [27], in NK cells [35], in myeloma [20], and melanoma [36]. The conclusion from all these observations is that the CD38/CD203a(PC-1)/CD73 pathway forms a network able to produce ADO, even if in a discontinuous fashion, meaning that not all the molecules need to be expressed by the individual cells, provided that they are operating in a closed environment.

At variance with the *Aplysia* ancestor, human CD38 enzyme is mainly a NAD^+^ glycohydrolase that leads to the generation of ADPR and NAM as products, accounting for >90% of the NADase activities. The cyclase function is present, but is approximately 100-fold lower than that of the hydrolase. A further limitation in the production of cADPR derives from the efficient conversion of cADPR to ADPR performed by the human enzyme. CD38 can also use NADP as a substrate and generate NAADP in the presence of nicotinic acid and at a suitable pH. This prevalently happens in the cytoplasm. Even if in limited amounts, cADPR and NAADP may thus be produced as a result of the enzymatic activities of CD38, while microenvironmental conditions (e.g., hypoxia, pH [37,38]) may shift the balance from one pathway to the other (see Section 8).

The CD38/CD203a(PC-1)/CD73 axis is operative in the niche with ADO production [20]. ADO levels were directly assayed in the niche, exploiting the unique opportunity provided by biopsies done in patients with monoclonal Ig components. The dependability of the ADO assay was achieved by defining the protocol of bone marrow (BM) plasma recovery and conservation. Further, it was also necessary to design an ad hoc HPLC test, to analyze qualitatively and quantitatively the components of NAD^+^ as metabolized by the converting ectoenzymes, knowing the short life of the final product [27].

**Figure 3:**
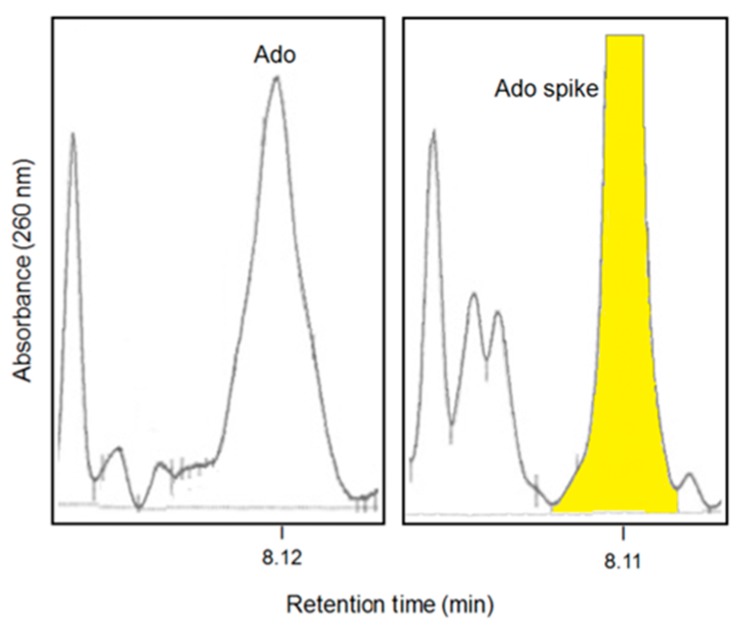
HPLC analysis of ADO (adenosine) content in BM (bone marrow) plasma aspirates from myeloma patients. In yellow is the ADO spiking.

The results indicate that ADO is significantly present in BM plasma [20]: the differences observed in quantitative amounts are under analysis to highlight the presence of links with disease stages (Figure 3).

**Figure 4:**
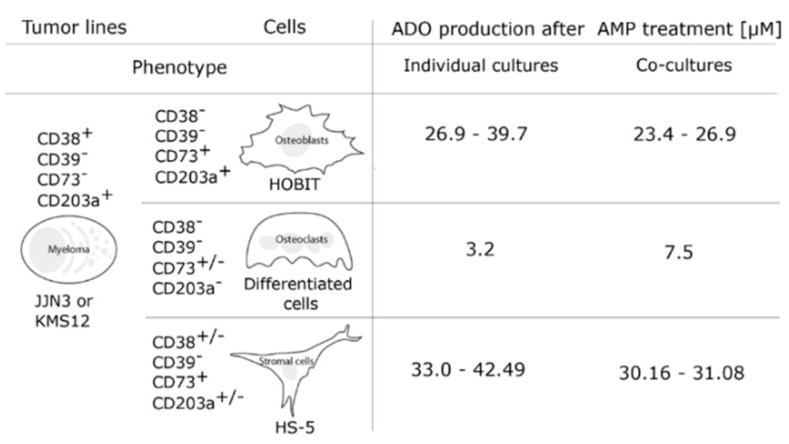
Production of ADO in co-cultures of a myeloma cell line, osteoblasts, osteoclasts (PBMC isolated from BM samples of MM (multiple myeloma) patients) and stromal cell line after AMP.

Which cells produce ADO and what conditions and synergies lead to the production of the nucleoside have only been partly defined. Preliminary answers to these questions were provided by experiments of co-cultures of twin combinations of myeloma cells with stromal cells, osteoblasts, and osteoclasts, respectively. The results indicate that ADO production appears mainly to be the consequence of interactions taking place between myeloma, on the one hand, and stromal cells and osteoclasts on the other (Figure 4). The main limitations of these experiments is the use of human cell lines and the current lack of blocking antibodies, monoclonal, or bispecific reagents [39].

## 8. Effects of pH on Adenosine Production in the Myeloma Niche

Hypoxia plays a key role in the reprograming of cancer cell metabolism. A well-known example is the shift from oxidative phosphorylation to glycolytic metabolism that promotes acidosis—due to the production of large amounts of lactic acid as an inevitable consequence of such metabolism—and the maintenance of redox homeostasis. The consequence for tumor cells is the establishment of an immune tolerance. Indeed, an increased production of ADO in the hypoxic microenvironment has been identified as a mechanism for immune modulation [40].

Acidification of the microenvironment is a conditional parameter of the MM niche: for this reason we analyzed a set of conditions able to drive and lead an *in situ* production of ADO.

**Figure 5:**
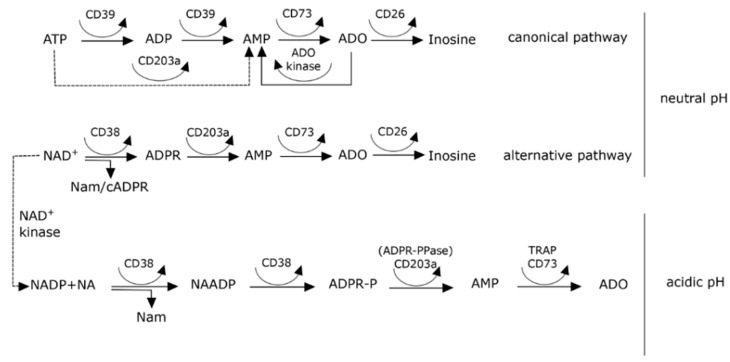
ADO production according to the environmental pH. Glossary: ADPR-P = ADP-ribose 2′-phosphate, ADPR-PPase = ADP-ribose pyrophosphatase, TRAP = tartrate-resistant acid phosphatase.

The generation of extracellular ADO from extracellular nucleotides occurs through the sequential enzymatic activity of the membrane-bound ecto-nucleotidases. The enzymatic activity of CD38 is highly dependent on pH: it is reasonable to assume that the *in vivo* activity of the enzyme may change according to the environment (Figure 5). Indeed, the cyclic adenine nucleotide cADPR, as well as the non-cyclic analog, are formed by CD38 under physiological conditions at neutral pH. NAADP is formed by a base-exchange mechanism on nicotinamide adenine dinucleotide phosphate (NADP) as a substrate, and where nicotinamide is exchanged with nicotinic acid. The latter must be present in excess and the reaction only proceeds at acidic pH.

The expression of CD73, which dephosphorylates AMP to ADO, is upregulated by HIF-1α [41]. However, the reduction of pH which occurs during aerobic glycolysis is followed by a marked inhibition of 5′-nucleotidase CD73 [42]. The effect is a reduction of ADO generation under such conditions. In such scenario it may be reasonable to speculate that a tartrate-resistant acid phosphatase (TRAP)—a nucleotidase active in acid environment—may efficiently cooperate in ADO production. ADO production under acidic conditions, *i.e.*, as in a bone resorptive environment typical of the MM niche, increases when myeloma cells are cultured in the presence of osteoclasts and after addition of the substrate NADP. Indeed, osteoclasts (CD38^+^/CD203a^+^/CD73^+^/TRAP^+^) obtained from differentiated PBMCs are able to metabolize the phosphorylated NAD^+^ at pH 5.5 in the presence of high concentrations of NA. The last step of the reaction is the production of NAADP, ADPR-P, AMP, and ADO.

The conclusion is that a local production of ADO may influence the immune response of the area. This action is facilitated by the physical constriction of the niche, while its short *in vivo* half-life is apparently unable to sustain long-lasting actions in the organism. Moreover, information gathered by pooling experimental data with empirical observations indicates that the hypoxic conditions present in the myeloma niche apparently favor the CD38/CD203a(PC-1)/CD73/TRAP pathway at the expense of the canonical axis (Figure 5).

## 9. The NAD^+^-Consuming CD38 Enzyme as a Local Modulator in the BM Myeloma Niche: Can Therapeutic mAb Confirm the Hypothesis?

**Figure 6:**
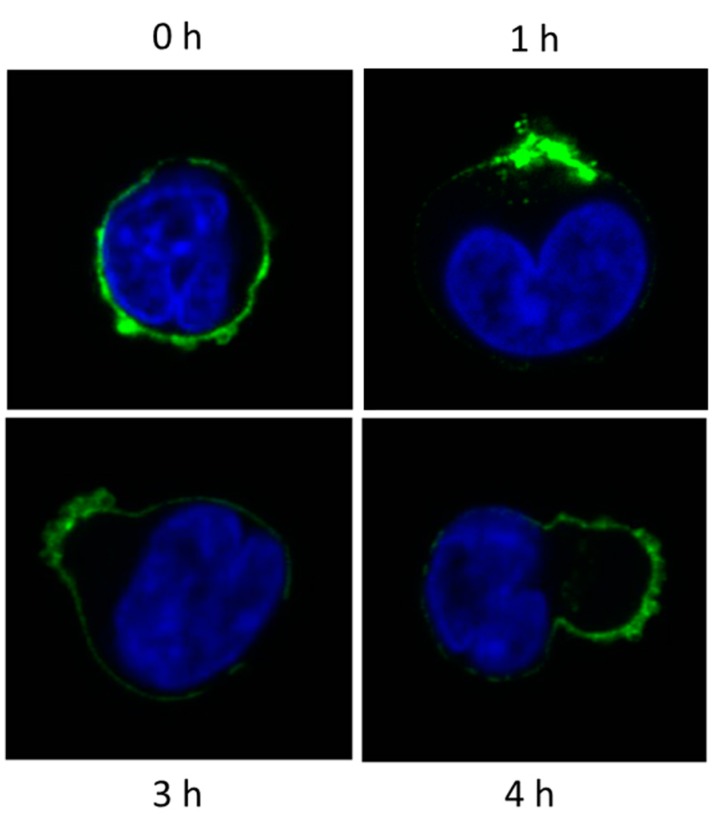
Confocal microscopy analysis of CD38/DARA (Daratumumab) interaction at 37 °C on the human BF01 myeloma line.

The original hypothesis was that CD38 plays a role in the generation of local tolerance by means of ADO production in closed systems [33]. This hypothesis was then expanded to include the possibility that similar mechanisms may be responsible for the widespread impairment of the immune system observed in myeloma patients. To investigate this issue, we documented the effects of the therapeutic mAb Daratumumab (DARA) on CD38. DARA is a human anti-human CD38 mAb in phase III clinical trials in patients with multiple myeloma. Ligation of CD38 by DARA led to important effects on the myeloma cytoskeleton, with a re-distribution of the CD38 molecules and formation of distinct polar aggregates (Figure 6). The behavior of DARA is extremely different from that observed using murine anti-human CD38 mAbs, marked by their tendency to internalize [43].

The results obtained using soluble DARA were confirmed by exposing myeloma cells to DARA immobilized on a battery of plastic-adhering CHO cells genetically modified to express a single human FcR, namely CD16, CD32A, CD32B, or CD64. This approach mimics the events that take place in the myeloma niche, where the therapeutic mAbs are prevalently bound by cells expressing FcRs [44]. The effects observed using immobilized DARA appeared as stronger than when using soluble DARA (data not shown).

## 10. Myeloma Cells Release Microvesicles upon DARA mAb Ligation

DARA ligation on myeloma cells was followed by a marked tendency to induce aggregation, polarization, and the subsequent release of microvesicles (MV). MV are vesicles of 100–1000 nm that originate directly from the plasma membrane. The process that leads to MV generation begins with formation of outward buds in specific sites of the membrane, followed by fission and the subsequent release of the vesicle into the extracellular space. MV are increasingly recognized as mediators of intercellular communication due to their capacity to merge with and transfer a collection of bioactive molecules to recipient cells [45]. The molecular and genetic contents of MV seem to be specific rather than a random sampling of all molecules expressed in the originating cell. MV release is reported as a dynamic process, dependent on both the cell type, activation status, and the environment from which the cells are released [46]. Compelling evidence suggests a role for tumor-derived MV-mediated modulation of cells within the tumor microenvironment of various malignancies, including lung and breast cancer, melanoma, and CLL [47]. Myeloma cells are also reported to release biologically active MV, contributing to cell proliferation [48]. We observed that the release of MV is a frequent event in the myeloma treated with therapeutic mAbs (Supplementary file).

The induction of MV may be relevant for *in vivo* myeloma therapy: MV are extrusions of the cell membrane and bear on their surface a set of molecules clustered in lipid microdomains, where the presence of CD38 has been confirmed.

The phenotype of MV is now under extended cytofluorimetric analysis by using mAbs specific for selected ectoenzymes that assist CD38 in regulating adenosine levels in the myeloma niche. Still to be answered is whether the molecules expressed on the surface of MV maintain their enzymatic functions, as is the case for exosomes [49].

### Fate of MV

The fate of the MV-bearing DARA on the surface is multiple: firstly, the MV may interact in the myeloma niche with the different cells present *in situ*. Another possibility is that the MV cross tissues and reach the blood stream, where they interact with the native cell populations. Due to the structural properties of the MV membrane, the lipid bilayer consents passive movement, even through tissues. This point is important in supporting the view of MV as minicellular signals delivering instructions at a distance from their place of origin [50]. Further support comes from the notion that the ectoenzymes analyzed are also molecules involved in cell migration or that may interact with countereceptors (e.g., CD31 for CD38) expressed by endothelial cells. Preliminary results show that MV-bearing DARA tend to cluster around NK cells and monocytes, both of which are rich in FcRs. The presence of DARA on their surface may act as a driving element: it is reasonable to think that FcR-expressing cells in tissues and peripheral blood or biological fluids may represent active and passive acceptors [51]. At the moment, one can only conclude that MV are associated with cell membranes through binding, likely mediated by FcRs.

## 11. Conclusions and Open Issues

The first part of this review provides an overview of the role played by NAD^+^ in human models. The almost exclusive focus on human models was imposed by the experience of our laboratory, along with compelling evidence coming from fields other than medicine. Indeed, NAD^+^ has been re-evaluated in different disease models and also as a target in potential therapeutic applications outside the most common inflammatory diseases [52,53].

In the context of diseases where the role of NAD^+^ is gaining momentum, we focused our attention on the human myeloma model, which satisfies several requisites for a disease to be informative for physiology. Multiple myeloma is a neoplastic expansion of plasma cells, whose plasma membranes express the highest epitope density of CD38, the ectoenzyme which consumes extracellular NAD^+^ with greatest efficiency. Further, the myeloma generally develops in bone marrow niches and is located in the closed system of the bone. Cells and fluids marking the events occurring within the niche are available and accessible for medical purposes. The characteristics of the molecule and its stability during the development of the disease render the molecule a useful target for therapeutic mAbs.

Our results indicate that CD38 is not merely an innocent marker, but that it plays a more direct role in tumor pathogenesis and likely clinical outcomes. Understanding the role of ADO in tumor growth and local immune defense is a matter of ongoing investigation and is particularly challenging because of the complexity of the disease itself. It would be of extreme interest to be able to use therapeutic mAbs to impair the ectoenzymatic function of the molecule, consequently blocking the inhibitory actions on Tregs and MDSC. Another important issue is to understand whether ADO levels in the BM niche can be associated with prognosis.

**Figure 7:**
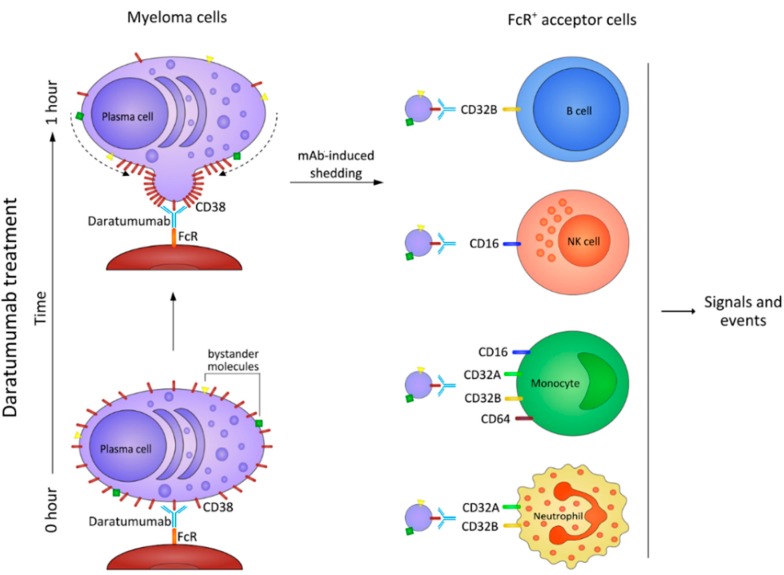
Events marking the interactions occurring *in vivo* after DARA treatment. Some steps are hypothetical.

Still to be analyzed in depth are the events following CD38 ligation and the induction of MV, which seem not to be just innocent bystanders in human myeloma [48]. It is possible that MV loaded with ectoenzymes leading to the production of ADO may trigger long-term responses, even after cessation of antibody treatment. A hypothesis already confirmed in animal models implies that tumors targeted by antibody therapy can induce the patient's immune system to generate an anti-tumor T cell memory response [51]. Another possibility under analysis is that MV loaded with ectoenzymes and transporting DARA might be able to reach the blood stream, to be taken up by monocytes and NK cells. This could lead to an increase in the lytic effects driven by NK cells and an interplay between effects mediated by CD32A (activatory signals) and CD32B (inhibitory signals) [44]. MV could also be internalized by FcR-expressing cells, with effects on both function and phenotype. A picture summarizing some results of the work is shown in Figure 7: some steps are still hypothetical.

## Supplementary Materials

Supplementary materials can be accessed at: http://www.mdpi.com/2073-4409/4/3/520/s1.
